# 

**Published:** 1917-08-11

**Authors:** 


### CONTENTS.

PAGE

**The Payment of Medical Officers in V.A.D. Hospitals** ... 367
**What Is Rat-Bite Fever?** ... 368
**Hospital and Institutional News** ... 369
Colonel Carter—The Inspection of Military Hospitals—Belgian Charity Funds—A Windfall for the Llanelly Hospital—The New Clinic in Golden Lane—German Soldiers in Withington Hospital —Mr. Barnes' Admission—The Ministry of Munitions and the Victims of the Explosion—The Welsh Sanatoria—A New Welsh Hospital Scheme—An Unexpected Gift—Sir Malcolm Morris and Huddersfield Infirmary—A Venereal Clinic at Exeter—Hospital Unrest in Swansea—A Children's Hospital in Difficulty—War Decreases Workmen's Contributions—Gas-Masks in Westminster Hall—A Vivisection Licence Revoked—A Record in Public Service—The Care of London's Mentally Deficient—Liverpool and Plague—Water Cisterns—The London Medical School Service—School Clinics for Durham—A Convalescent Home for Munition Workers—Baby Welfare in Ontario—Child Welfare in Liverpool —Raid Arrangements—A Staffing Problem—Lunatics in Office—Emergency Medicine—This Week's Drug Market—To Our Readers.
**The Worst War of the World** ... 375
II. Its Third Anniversary in Westminster Abbey. The Abbey Service.
**War Matinees and the Public Health** ... 377
Essential Defects of the Theatre and Cinema.

PAGE

**Initis or Nutrition and Exercises** ... 378
**Physical Orthopædics...** ... 379
A Grave National Deficiency.—I.
**The Matrons' and Sisters' Department** ... 381
Matrons in Council.—Some Return Sparks: A Matron's View
Round the Hospitals.
The Queen's Indisposition—New War Hymn: "O Valiant Hearts"—German Pirates, Nurses and Hospital Ships—Mrs. Minet's Three Medals for Nurses—Miss Rundle and Councillor Sheardown—The "Choker" Collar: Another View.
**The Hygiene of Play...** ... 384
**Metropolitan Hospital Sunday Fund** ... 385
Distribution Committee's Report and Awards.
Awards Recommended by the Committee of Distribution for the Year 1917.
**New War Hymn: "O Valiant Hearts"** ... iv
**Medical Appointments** ... iv
**Matrons' and Sisters' Appointments** ... iv
**Parliament and the Profession** ... iv
**The Institutional Worker** ... (Supl.) 1
The Control of the Laundry: Answer to July Question.
Question for August.
Questions Invited.
Enquiries and Answers.
Employment and Training.
The Housekeepers' Department.

